# Characterization of Adsorbents for Cytokine Removal from Blood in an* In Vitro* Model

**DOI:** 10.1155/2015/484736

**Published:** 2015-12-07

**Authors:** Stephan Harm, Franz Gabor, Jens Hartmann

**Affiliations:** ^1^Department for Health Sciences and Biomedicine, Danube University Krems, Dr.-Karl-Dorrek-Straße 30, 3500 Krems, Austria; ^2^Department of Pharmaceutical Technology and Biopharmaceutics, University of Vienna, Althanstraße 14, 1090 Vienna, Austria

## Abstract

*Introduction*. Cytokines are basic targets that have to be removed effectively in order to improve the patient's health status in treating severe inflammation, sepsis, and septic shock. Although there are different adsorbents commercially available, the success of their clinical use is limited. Here, we tested different adsorbents for their effective removal of cytokines from plasma and the resulting effect on endothelial cell activation.* Methods*. The three polystyrene divinylbenzene (PS-DVB) based adsorbents Amberchrom CG161c and CG300m and a clinically approved haemoperfusion adsorbent (HAC) were studied with regard to cytokine removal in human blood. To induce cytokine release from leucocytes, human blood cells were stimulated with 1 ng/ml LPS for 4 hours. Plasma was separated and adsorption experiments in a dynamic model were performed. The effect of cytokine removal on endothelial cell activation was evaluated using a HUVEC-based cell culture model. The beneficial outcome was assessed by measuring ICAM-1, E-selectin, and secreted cytokines IL-8 and IL-6. Additionally the threshold concentration for HUVEC activation by TNF-*α* and IL-1*β* was determined using this cell culture model.* Results*. CG161c showed promising results in removing the investigated cytokines. Due to its pore size the adsorbent efficiently removed the key factor TNF-*α*, outperforming the commercially available adsorbents. The CG161c treatment reduced cytokine secretion and expression of cell adhesion molecules by HUVEC which underlines the importance of effective removal of TNF-*α* in inflammatory diseases.* Conclusion*. These results confirm the hypothesis that cytokine removal from the blood should approach physiological levels in order to reduce endothelial cell activation.

## 1. Introduction

Sepsis is a systemic inflammatory response syndrome (SIRS) that results from the body's innate immune response triggered by any of the several infectious stimuli. Lipopolysaccharides (endotoxins), peptidoglycan, flagellin, lipoteichoic acid from bacteria, mannan from fungi, and other antigens from infectious agents stimulate monocytes and macrophages to release tumour necrosis factor alpha (TNF-*α*) as well as interleukins 1 and 6 (IL-1, IL-6) into the circulation [1–4]. These again activate additional proinflammatory pathways within endothelial cells and leukocytes. A very high and uncontrolled release of proinflammatory cytokines also stimulates leukocytes to release anti-inflammatory mediators and transforming growth factor-beta, which inhibit the synthesis of proinflammatory cytokines and exert direct anti-inflammatory effects on monocytes, macrophages, and endothelial cells [5]. In many cases progress (the further course) of the disease will lead either to an unbalanced coexistence of pro- and anti-inflammatory mediators (mixed antagonistic response syndrome) or to an excess of anti-inflammatory cytokines which end up in immunosuppression. This so-called sepsis-induced “immunoparalysis” is characterized by restricted innate and adaptive immune responses, including enhanced apoptosis and dysfunction of lymphocytes and impaired phagocyte functions [6]. A sensitive balance between proinflammatory and anti-inflammatory response is necessary for cytokine release to achieve homeostasis. Attempts were made to restore the cytokine imbalance by using anticytokine monoclonal antibodies. These attempts, where particular cytokines were blocked, yielded no clinically detectable benefits but indicated that the modulation of several cytokines at the same time to reach rather physiological blood levels may help to achieve homeostasis [7]. Consequently, extracorporeal blood purification (EBP) techniques were applied to modulate pro- and anti-inflammatory cytokines of sepsis patients. Currently, there are four main techniques in clinical use for cytokine removal: high-flux hemofiltration, high cutoff membranes, adsorption techniques, and combined plasma filtration adsorption [7]. A hemoperfusion cartridge that is used for cytokine removal in intensive care medicine is the Cytosorb cartridge, which is filled with 300 mL hemadsorption beads [8]. Cytosorb hemadsorption beads are porous polystyrene-divinylbenzene (PS-DVB) particles coated with biocompatible polyvinylpyrrolidone exhibiting 450 *μ*m average particle diameter and 0.8–5 nm pores [9, 10]. Another device for cytokine removal is the Coupled Plasma Filtration Adsorption (CPFA). CPFA is an extracorporeal therapy that was developed and patented by Bellco for the treatment of patients with multiorgan failure or sepsis. CPFA combines plasma sorption and hemofiltration for cytokine elimination in patients' blood. The unspecific removal of inflammatory mediators is achieved by an Amberchrom adsorbent [11]. This hydrophobic polystyrene resin with an average pore size of 30 nm has a high affinity and capacity for many cytokines and mediators [12]. Both adsorbents were clinically tested and capable of decreasing proinflammatory cytokines significantly, but a reduction of mortality in patients with septic shock was not observed [13, 14]. Probably the removal rate of cytokines was not sufficient to reach homeostasis. In a previous study conducted by our group, the optimal pore size for cytokine removal was investigated [15] and revealed that the Amberchrom CG161c, a neutral PS-DVB based adsorbent with 15 nm pores, shows promising results for cytokine removal from human plasma. The aim of this study was to compare, by* in vitro* experiments using human plasma, the capability of cytokine removal between the new CG161c adsorbent and the two PS-DVB based cytokine adsorbents available for clinical use. Furthermore, the consequence of the level of cytokine removal achieved by each adsorbent on endothelial cell activation was tested using human umbilical vein endothelial cells (HUVECs).

## 2. Materials and Methods

### 2.1. Materials

The clinically approved hemoperfusion adsorbent for cytokine removal (HAC) was obtained from Euromed (Euromed GmbH, Vienna, Austria) and the two Amberchrom adsorbents CG300m and CG161c were provided by Dow Chemical (Philadelphia, PA, USA). Tetrahydrofuran, toluene, and polystyrene standards for inverse size exclusion chromatography (iSEC) were purchased from Sigma-Aldrich (St. Louis, MO, USA) and ethanol was obtained from VWR (Vienna, Austria). Blood bags were ordered from the Red Cross (Vienna, Austria) and the Bio-Plex cytokine array was purchased from Biorad (Biorad, Vienna, Austria). Recombinant TNF-*α* and IL-1*β* were obtained from R&D Systems (Minneapolis, MN). Hank's Balanced Salt Solution, cell culture medium M199, penicillin, streptomycin, fetal bovine serum (FBS), endothelial cell growth supplement (ECGS), and HEPES buffer were purchased from Sigma-Aldrich (St. Louis, MO, USA).

### 2.2. Adsorbent Characterization

#### 2.2.1. SEM

The structural characteristics and accessible pore size of each adsorbent were determined by scanning electron microscopy (SEM) and inverse size exclusion chromatography. The adsorbent particles were washed with pure ethanol and dried at 100°C for 12 hours. The particles were then sputtered with gold (Q150R ES, QUORUM) and imaged by SEM (TM-1000, Table Microscope, Hitachi).

#### 2.2.2. Particle Size

Particle size distributions of the microspheres were determined by laser-light scattering (Mastersizer 2000, Malvern Instruments, Malvern, UK). Approximately 500 *μ*L of microspheres suspension was dispersed in 100 mL distilled water and sonicated to avoid agglomeration of particles during measurements. The particle size distribution results are volume based.

#### 2.2.3. Pore Size

Inverse size exclusion chromatography was used to determine the accessible pore size and intraparticle porosity of each adsorbent based on the retention of toluene and polystyrene standards with molecular masses between 0.5 and 1,000 kDa. For this purpose, each adsorbent was flow packed in 0.46 × 15 cm HPLC columns from Grace Davison Discovery Sciences (GRACE). A Waters HPLC System (Milford, USA) with a Waters 2487 UV detector was used to determine the retention volume of individual standards after injection of 20 *μ*L samples containing 10 mg/mL polystyrene at a flow rate of 0.5 mL/min. The retention volume of each polystyrene standard was experimentally determined and the SEC distribution coefficient has been calculated according to the following:

(1) $$\begin{array}{c} K_{d}=\frac{V_{R}-V_{0}}{V_{T}-V_{0}}, \end{array}$$

where *V*_*R*_ is the retention volume, *V*_0_ is the interparticle void volume, and *V*_*T*_ is the total mobile phase volume. The mobile phase was represented by Tetrahydrofuran. Toluene was used as a small molecule tracer and acetonitrile only for washing. *K*_*d*_ values range between 0, for a compound that is excluded completely corresponding to polystyrene with a molecular mass of 1,000 kDa, and 1, for compounds able to access and permeate the total pore volume represented by toluene with a molecular mass of 92 Da. Since (*V*_*T*_ − *V*_0_) represents the intraparticle mobile phase volume, *K*_*d*_ represents the extent of permeation for molecules into the pore volume of the stationary phase. The following correlation was used in order to interrelate the molecular mass *M*_*W*_ of a polystyrene sample and the size of the pores (Φ) from which it is excluded:

(2) $$\begin{array}{c} M_{W}=2.25\times \Phi^{1.7}, \end{array}$$

where the pore size diameter is given in Å [16, 17].

The adsorbent porosity *ε*_*P*_ was calculated from the following [18, 19]:

(3) $$\begin{array}{c} \varepsilon_{P}=\frac{V_{T}-V_{0}}{V_{B}-V_{0}}, \end{array}$$

where *V*_*B*_ is the column bed volume.

The pore volume (*V*_*P*_) of the adsorbent materials was calculated according to

(4) $$\begin{array}{c} V_{P}=V_{T}-V_{0}. \end{array}$$

### 2.3. An* In Vitro* Sepsis Model

**Figure 1:**
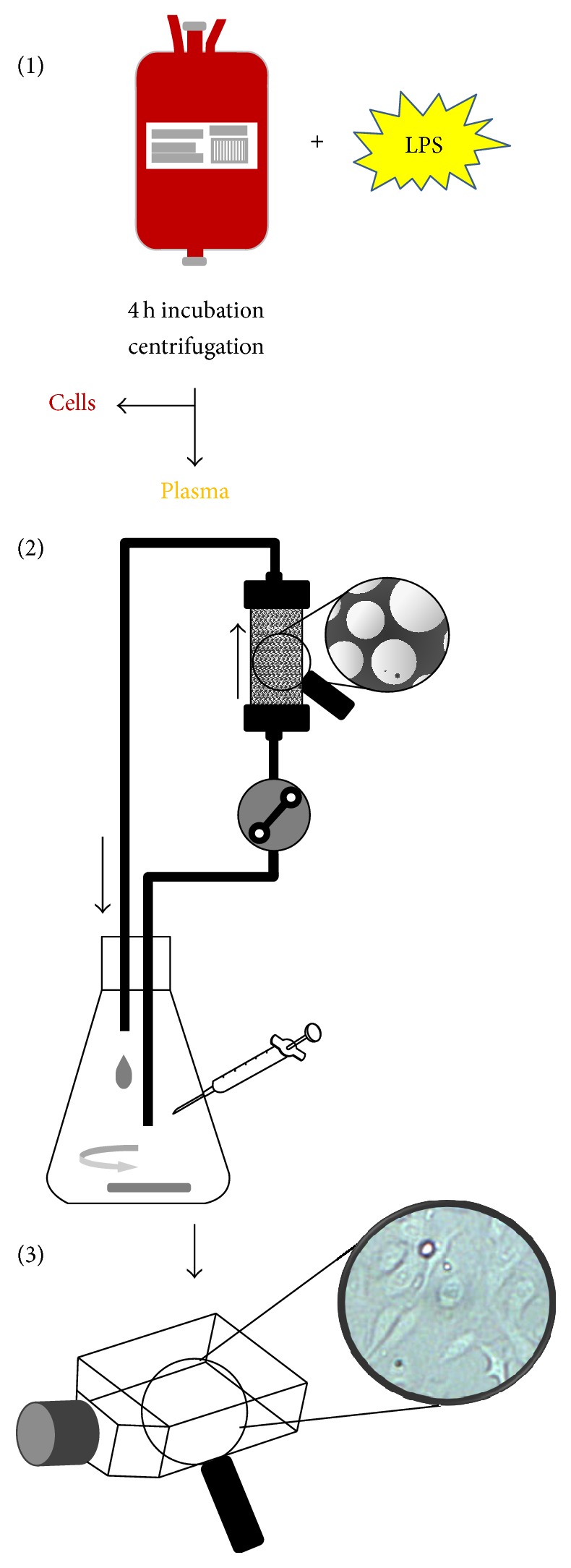
Schematic procedure of the experiments. The experiments were conducted in three parts: (1) blood stimulation and centrifugation, (2) adsorption experiments by a dynamic model, and (3) cell culture model with HUVEC.

The three PS-DVB based adsorbents Amberchrom CG161c, Amberchrom CG300m, and HAC were studied in a dynamic model with regard to cytokine removal in human plasma. Furthermore, the effect of cytokine removal on endothelial cell activation was evaluated using human umbilical vein endothelial cells (HUVECs). This model comprises three steps: whole blood stimulation, the adsorption study in a dynamic model, and the cell culture model (see Figure 1).

#### 2.3.1. Whole Blood Stimulation

Blood bags containing between 400 and 500 mL fresh donated blood were ordered from the Red Cross (Vienna, Austria). The overproduction of cytokines by leucocytes was induced by stimulating human blood with 1 ng/mL LPS from* E. coli* (Sigma, St. Louis, MO, USA) at 37°C for 4 hours. The plasma, including the inflammatory mediators, was separated by centrifugation at 3000 ×g for 10 min and then stored at −80°C until adsorption experiments were performed in a dynamic model.

#### 2.3.2. Adsorption Studies in a Dynamic Model

The dynamic model consists of a commercially available 5 mL Rezorian cartridge (Sigma, St. Louis, MO, USA) packed with 5 mL of adsorbent material. The bead volume of the cartridge was downscaled (approximately 60x) in comparison to the 300 mL cartridge which is normally used clinically for the HAC device. The recirculation reservoir volume, 60 mL, and flow rates, 1 mL/min (55 cm/h), used in the experiments were also scaled down from clinical hemadsorption, 100 to 300 mL/min (212–635 cm/h), and a total blood volume of 4 to 6 liters in the average adult, using this factor (see Figure 1). A circuit with an empty cartridge acted as a control. The experiment was carried out for 6 hours at 37°C, and samples were taken hourly and stored at −80°C until cytokine quantification using the Bio-Plex cytokine array and the cell culture model for endothelial cell activation were performed. In order to ensure the plasma stability during the experiment, albumin, total protein, antithrombin III, protein C, and fibrinogen were measured at the beginning and at the end of the experiment using a Hitachi/Roche 902 automated analyser with the according test kits (Roche, Penzberg, Germany).

#### 2.3.3. Endothelial Cell Activation

*(1) Cell Culture*. The effect of cytokine removal on endothelial cell activation was evaluated using a cell culture model with HUVEC. The beneficial outcome was assessed by measuring the adhesion molecules ICAM-1 and E-selectin and an array of secreted cytokines after incubation of HUVEC with 10% of plasma from the adsorption experiments. Primary HUVECs were isolated from umbilical cord veins provided by the local hospital (LKH-Krems, Austria) after informed consent of the donors and stored at 4°C in sterile HBSS. HUVECs were isolated according to Marin et al. with minor changes [20]. Cannulated umbilical veins were perfused with M199 containing 0.02 M HEPES and 100 mM penicillin-streptomycin (M199/HEPES/PS) at 37°C to remove the blood. The veins were filled with dispase (BD Biosciences Europe, Vienna, Austria) and incubated at 37°C for 15 min. After incubation, the dispase solution containing the HUVEC was collected by perfusion of the cord with basal medium (M199/HEPES/PS). The cells were collected by centrifugation at 500 ×g for 5 min and resuspended in growth medium (M199/HEPES/PS containing 20% FBS, 15 IU/mL heparin, and 10 mg/mL ECGS) and transferred to a 75 cm^2^ cell culture flask. One day after isolation, cells were washed with basal medium and supplied with fresh growth medium. Isolated HUVECs were used between passages 4 and 7 for the assays.

*(2) Stimulation of HUVEC*. HUVECs (8.5 × 10^5^) were seeded in 25 cm^2^ cell culture flasks with 5 mL growth medium and incubated for 24 hours at 37°C and 5% CO_2_. The cell activation tests were performed after cell vitality and near confluency were confirmed by microscopy, as follows: plasma samples from the adsorption experiments were thawed and diluted 1 : 10 with 5 mL of basal medium. The HUVEC monolayer was washed once with basal medium and the sample medium was added to the corresponding cell culture flask. The cells were incubated with the sample medium and control medium (basal medium including 10% native plasma) for 16 hours at 37°C and 5% CO_2_ atmosphere. After incubation the supernatants were centrifuged for 10 min at 1000 g, aliquoted, and stored at −80°C until cytokine analysis by the Bio-Plex cytokine array. The cells were washed with 3 mL of ice-cold PBS and detached with 1.5 mL 0.02% EDTA per flask. After addition of 3 mL PBS, cells were pelleted at 500 ×g for 5 min and used for flow cytometry analysis.

*(3) Flow Cytometry Analysis*. The detached cells were counted and aliquots of 2.5 × 10^5^ cells per sample were prepared in FACS tubes. The cells were washed with ice-cold PBS and stained by incubation with FITC-conjugated anti-CD31, PE-conjugated anti-ICAM-1, PE-Cy5-conjugated anti-E-selectin (BD, Franklin Lakes, NJ, USA), or the corresponding control antibodies for 30 min on ice in the dark. All antibodies were from the IgG isotype. After two further washing steps with PBS, cells were analysed on a flow cytometer (Cytomics FC 500 MPL, Beckman Coulter, CA, US), using the FlowJo 7.6.5 software (Tree Star Inc., Ashland, OR, USA).

### 2.4. TNF-*α* and IL-1*β* Dependent Activation of HUVEC in the Cell Culture Model

To evaluate the level, to which the cytokines have to be lowered by any extracorporeal treatment, for preventing or reducing the endothelial cell activation, a separate experiment was performed. Heparinized (5 IU/mL) human plasma with different recombinant TNF-*α* and IL-1*β* concentrations (0, 50, 100, 500, 1000, 5000, and 10000 pg/mL) was used in our cell culture model to determine their threshold level for endothelial cells activation. The HUVECs were cultivated with sample medium (as described above) including 10% of spiked plasma which leads to a tenfold dilution of the spiked recombinant cytokines. After 16 hours of incubation, the supernatants were aspirated, centrifuged for 10 min at 1000 ×g, aliquoted, and stored at −80°C until IL-8 and IL-6 were quantified by the Bio-Plex cytokine array. To verify the expression of the adhesion molecules ICAM-1 and E-selectin, the HUVECs were washed and analysed by flow cytometry as described above.

## 3. Results and Discussion

### 3.1. Adsorbent Characterization

#### 3.1.1. SEM, Particle Size, and Inverse Size Exclusion

**Figure 2:**
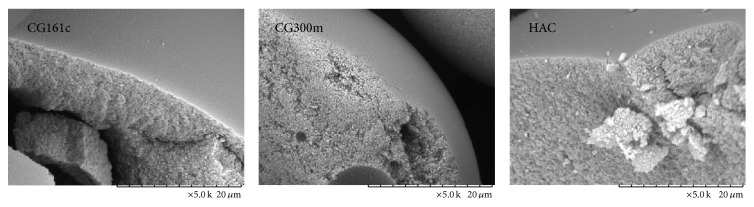
Imaging and particle size of the used adsorbents. SEM images at 5000x magnification and particle size distribution using laser-light scattering of the three tested adsorbents.

SEM of the manually cracked particles illustrates that the outer thin shell of the adsorbent particles acts as a molecular sieve for entering the inner surface, which is the adsorbent surface for the target molecules (see Figure 2 and Table 1). The *K*_*d*_ values obtained for toluol and polystyrene probes from the iSEC experiments are shown in Table 2. Complete molecular exclusion is achieved when the *K*_*d*_ value reaches zero at a certain molecular weight. For the largest pore size, an acceptable *K*_*d*_ was defined with a value of 0.1, which means that molecules with a *K*_*d*_ of 0.1 are allowed to pass through the outer pore shell and reach the inner adsorbent surface. As shown in Table 2, *K*_*d*_ approaches 0.1 at a pore size between 10.0 and 16.2 nm for CG161c, 20.6 and 26.0 nm for CG300m, and 7.6 and 10.0 nm for HAC. The porosities *ε*_*P*_ (see Table 2) of the three tested adsorbent particles were similar and always above 80%. HAC was found to have the highest porosity at 86.6% followed by CG161c, 86.2%, and CG300m, 82.3%.

### 3.2. Adsorption Studies in Dynamic Model

**Figure 3:**
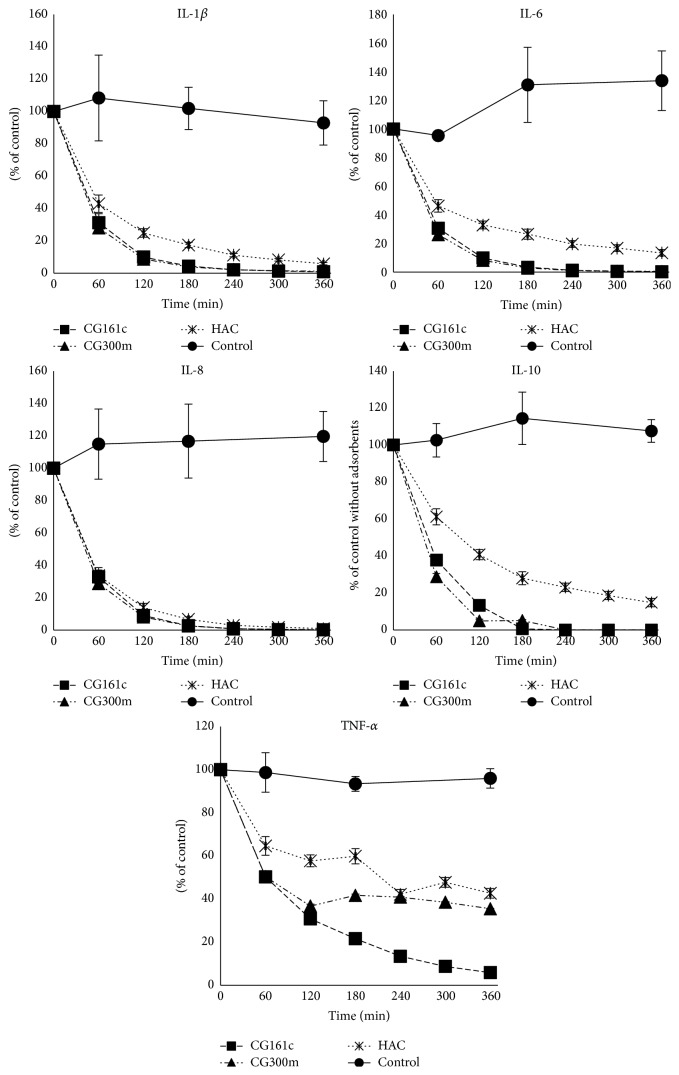
Cytokine removal in the dynamic model. Cytokine levels (TNF-*α*, IL-1*β*, IL-6, IL-8, and IL-10) in the plasma pool during 6 hours of treatment with the three tested adsorbents in the dynamic model. The results are shown in mean ± SD.

The ability of the adsorbents to remove cytokine was investigated in dynamic model experiments using inflammatory mediator rich human plasma obtained after whole blood stimulation. The concentrations of the following cytokines were measured hourly over a 6-hour period: TNF-*α*, IL-1*β*, IL-6, IL-8, and IL-10. These cytokines are considered to be key factors as well as markers in inflammation [21–23]. However, there are many other cytokines and mediators involved in this complex and dynamic process. The interleukins were efficiently removed by both CG161c and CG300m at comparable levels. HAC performed consistently worse than the other adsorbents for all tested cytokines. Sufficient TNF-*α* removal could only be observed in case of CG161c (Figure 3 and Table 3) with a removal rate of 94.3 ± 0.23%. The two commercially available cytokine adsorbents offered limited removal of TNF-*α*: 63.5 ± 0.5% for CG300m and 53.4 ± 6.8% for HAC. The molecular mass of TNF-*α* ranges from 17 to 51 kDa depending on oligomerization, that is, monomer, dimer, or trimer. The homotrimer is the most active form of TNF-*α*, which is the largest cytokine with respect to the crystal structure and viscosity radius [15]. Because of the large size of the trimer, the removal of TNF-*α* from the bloodstream represents a considerable significant challenge. The mechanism of adsorption of the three adsorbents under investigation is the same. The target molecules have to enter the pores of the outer surface to reach the inner surface composed of PS-DVB copolymer, where they will be adsorbed. The particle size, the pore size (determined by iSEC), and the blood compatible outer PVP layer in case of HAC are the only differentiation factors suggesting that the reduced adsorption is primarily due to the different pore sizes of the adsorbents. When the pores are too small, TNF-*α* cannot enter the adsorbent beads to be immobilized at the inner surface. Contrariwise, if the pore size is too large, bound TNF-*α* may be replaced by high molecular weight plasma proteins and plasma lipids due to their high binding affinity via hydrophobic interactions according to the Vroman effect [24]. There was no significant change in the parameters (albumin, total protein, antithrombin III, protein C, and fibrinogen) which were observed in order to ensure plasma stability during the experiment (data not shown).

### 3.3. Cell Culture Model

**Figure 4:**
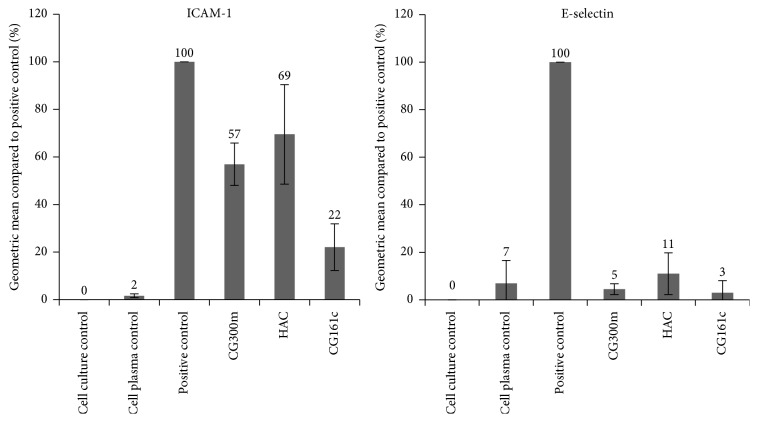
ICAM-1 and E-selectin expression by HUVEC. The effect of cytokine removal on endothelial cell activation was assessed by measuring the expressed adhesion molecules ICAM-1 and E-selectin after incubation of HUVECs with 10% of sample plasma from the adsorption experiments. The results are shown in mean ± SD.

The endothelium takes part in the regulation of numerous physiological functions and lies at the interface of circulating blood and the vessel wall. Under physiological conditions, it is responsible for anticoagulant and antiadhesive properties and it regulates vasomotor tone and vascular homeostasis. Endothelial dysfunction has been associated with many pathophysiological processes, such as inflammation and oxidative stresses. Endothelial cells are precociously exposed to circulating signalling molecules and physical stresses, like in sepsis and septic shock [25]. It is well known that sepsis in humans is associated with activation of the endothelium as evidenced by increased levels of expressed ICAM-1 and E-selectin and secreted cytokines/chemokines such as IL-6 and IL-8. To test whether cytokine removal has a positive effect on endothelial cell activation, the treated plasma derived from the adsorbent experiments was used to stimulate HUVEC. The results of the flow cytometry analysis indicate that the CG161c adsorbent is most effective at reducing the expression of cell adhesion molecules by HUVEC. The expression of the adhesion molecule E-selectin could effectively be suppressed by all three adsorbent treatments. A marked difference, however, was observed in case of ICAM-1 with a highly reduced expression to 22 ± 10% for CG161c and a moderate beneficial effect for CG300m (57 ± 9%) as well as for HAC (69 ± 21%) compared to the untreated cytokine rich plasma. It is well documented that ICAM-1 expression in vascular endothelium can be induced by IL-1 and TNF-*α* [26]. This fact should be considered when the results of TNF-*α* removal (Figure 3) and the resulting ICAM-1 expression (Figure 4) are compared. It confirms that the lower the TNF-*α* content in plasma, the lower the ICAM-1 expression on HUVEC's surface. Thus, only an effective removal of cytokines to a physiological concentration in plasma, which are usually below 100 pg/mL [27], can significantly reduce endothelial cell activation.

**Figure 5:**
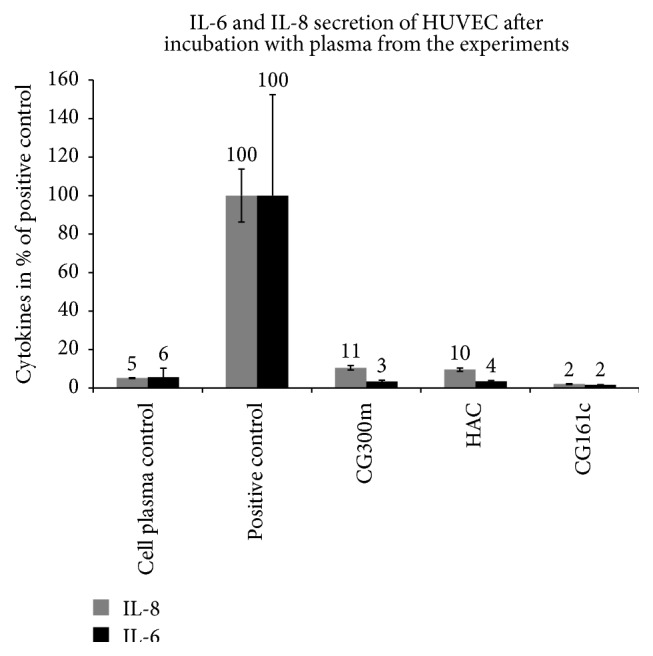
IL-6 and IL-8 secretion by HUVEC. The effect of cytokine removal on endothelial cell activation regarding IL-8 and IL-6 secretion after 16 h incubation of HUVEC with 10% of sample plasma from the adsorption experiments. Untreated plasma (empty cartridge without adsorbent) acts as positive control. The results are shown in mean ± SD.

**Figure 6:**
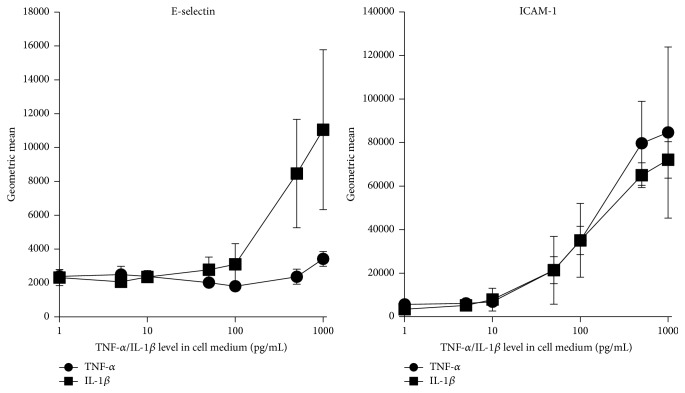
E-selectin and ICAM-1 expression of HUVEC as a function of TNF-*α* or IL-1*β* level. ICAM-1 and E-selectin expression by HUVEC after 16-hour incubation in cell media with 10% of plasma spiked with increasing amounts of TNF-*α* or IL-1*β* (mean ± SD, *n* = 3).

A similar effect was observed when the secreted cytokines of HUVEC were determined. The plasma treated with the CG161c adsorbent elicits the lowest IL-6 and IL-8 levels in cell culture but also the other two tested adsorbents provoke a high reduction in cytokine release compared to untreated plasma (Figure 5 and Table 4). Makó et al. reported that the expression of E-selectin, IL-6, and IL-8 was induced most efficiently by IL-1*β*, while that of LPS and TNF-*α* was less efficient, and ICAM-1 expression was not different between stimuli [28]. Our findings are in agreement with those of Makó et al. (see Figure 6), because IL-1*β* was removed very efficiently by all tested adsorbents; also the ICAM-1 expression as well as IL-6 and IL-8 secretion from the HUVEC was reduced.

### 3.4. TNF-*α* and IL-1*β* Dependent Activation of HUVEC in the Cell Culture Model

**Figure 7:**
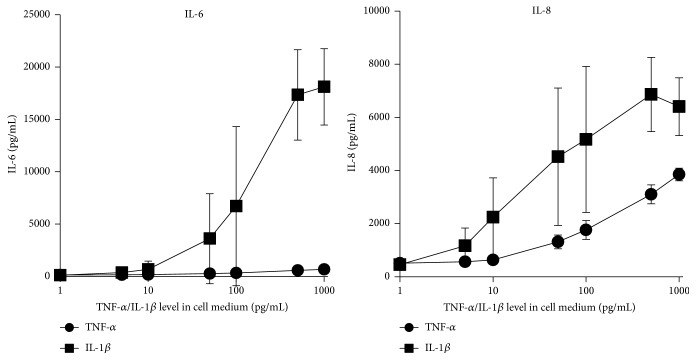
TNF-*α* or IL-1*β* dose-dependent production of IL-6 and IL-8 by HUVEC. IL-6 and IL-8 secretion by HUVEC after 16-hour incubation in cell media with 10% of plasma spiked with increasing amounts of TNF-*α* or IL-1*β* (mean ± SD, *n* = 3).

Endothelial cells are activated primarily by the two cytokines TNF-*α* and IL-1*β* [28, 29]. In our cell culture model the threshold concentrations of IL-1*β* and TNF-*α* for HUVEC activation were between 10 and 50 pg/mL (see Figure 6) regarding IL-8 and IL-6 secretion as well as ICAM-1 expression (Figures 6 and 7). E-selectin expression was induced by low IL-1*β* concentration (50 pg/mL) in contrast to TNF-*α* which activates the expression of E-selectin not below 500 pg/mL. Thus the three tested adsorbents which removed IL-1*β* very efficiently also were able to reduce the expression of E-selectin in contrast to ICAM-1 expression which was only suppressed by the CG161c treatment because of efficient TNF-*α* removal. These results confirm the assumption that it is not sufficient merely to reduce the cytokine levels, for example, by EBP. The cytokine levels have to be reduced to physiological levels in order to prevent endothelial cell activation. Based on their molecular size, especially trimeric TNF-*α* with 52 kDa, the cytokines are not able to rapidly cross the usually applied dialysis membranes. Consequently, an effective removal of a wide array of cytokines from the plasma cannot be achieved using only membrane based technologies like high-volume hemofiltration and high-cutoff hemodialysis or hemofiltration. This can only be realised by using adsorption techniques or by a combination of adsorption and membrane technologies.

## 4. Conclusion

Cytokines are considered to be targets that have to be modulated in order to improve the patient's health in case of severe inflammation, sepsis, and septic shock. Although there are different adsorbents commercially available, their clinical utility is limited [30]. In order to suppress systemic effects in these disease patterns, effective removal of cytokines below a critical threshold is necessary. The three PS-DVB based adsorbents Amberchrom CG161c, Amberchrom CG300m, and HAC were studied with regard to cytokine removal capacity from human plasma. The new PS-DVB based cytokine adsorbent CG161c exhibited promising results in terms of all tested cytokines. Especially in case of removing the key factor TNF-*α*, it outperforms commercially available adsorbents such as HAC or CG300m due to its optimized pore size. With respect to endothelial cell activation, the CG161c treatment highly reduced cytokine secretion and expression of cell adhesion molecules in HUVEC, which emphasizes the importance of the effective removal of TNF-*α* in inflammatory diseases when using a cytokine adsorber. A successful sepsis treatment strategy regarding effective cytokine modulation may use a combination of membrane and adsorption based technique. A promising adsorbent for such a blood purification device could be the CG161c adsorbent. However, the findings here are based on* in vitro* studies and are not yet confirmed by clinical data.

## Key Messages

(i) The Amberchrom adsorbent CG161c is promising for cytokine removal from human plasma compared to other tested cytokine adsorbents.
(ii) The threshold concentrations of TNF-*α* and IL-1*β* for HUVEC stimulation are below 50 pg/mL.
(iii) Cytokines circulating in the blood should be modulated to physiological levels during treatment of sepsis in order to reduce endothelial cell activation.

## Figures and Tables

**Table 1:** 

Adsorbent	*D*(0.1)	*D*(0.5)	*D*(0.9)	Vol. weighted mean *D* [*μ*m]
	[*μ*m]	[*μ*m]	[*μ*m]	
CG300m	60	82	112	84
CG161c	86	117	158	120
HAC	363	492	656	504

**Table 2:** Summary of inverse size exclusion chromatography. The pore size of the adsorbents was determined by SEC using polystyrene standards. *R*_*S*_: stokes radius;  *K*_*d*_: SEC distribution coefficient.

*M*_*r*_	*R*_*S*_ [nm]	*K*_*d*_	*K*_*d*_	*K*_*d*_
		CG161c	CG300m	HAC
92	0.18	1.00	1.00	1.00
570	0.51	0.82	0.85	0.56
1920	1.05	0.67	0.76	0.29
3460	1.48	0.59	0.70	0.21
9630	2.71	0.41	0.60	0.11
17300	3.82	0.29	0.53	0.10
27500	5.01	0.15	0.44	0.08
62300	8.11	0.02	0.26	0.04
96000	10.46	0.00	0.13	0.03
139000	13.00	0.00	0.07	0.02
319000	21.19	0.00	0.01	0.01
524000	28.37	0.00	0.00	0.00
925000	39.63	0.00	0.00	0.00

		CG161c	CG300m	HAC

*V*_*T* (Toluol)_ [mL]		2.30	2.25	2.31
*V*_0 (PS 1000 kDa)_ [mL]		1.06	1.14	1.13
*V*_*B* (column)_ [mL]		1.792	1.792	1.792
*V*_*P*_ [mL]		1.23	1.11	1.18
*ε*_*P*_		86.2	82.3	86.6
^*∗*^iSEC pore radius *r* [nm]		5.0 < *r* < 8.1	10.3 < *r* < 13.0	3.8 < *r* < 5.0
^+^Pore radius [nm]		7.5	15	2.5

^*∗*^ *K*_*d*_ > 0.1.

^+^Manufacturer data.

**Table 3:** Cytokine levels of treated plasma. Mean cytokine concentration ± SD (*n* = 3) in plasma after 6 hours of treatment with different adsorbents in the dynamic model. Plasma from LPS treated blood circulating through an empty cartridge acted as control.

	TNF-*α* [pg/mL]	IL-1*β* [pg/mL]	IL-6 [pg/mL]	IL-8 [pg/mL]	IL-10 [pg/mL]
Control	3102 ± 533	830 ± 190	24273 ± 13446	4837 ± 2300	51 ± 9
CG161c	177 ± 7	7 ± 1	59 ± 18	8 ± 7	<2
CG300m	1131 ± 16	10 ± 1	65 ± 3	10 ± 6	<2
HAC	1445 ± 212	45 ± 3	2587 ± 1254	60 ± 52	7 ± 4

**Table 4:** IL-6 and IL-8 secretion by HUVECs. Mean IL-6 and IL-8 secretion ± standard deviation (*n* = 3) of HUVECs after a 16-hour treatment with cell media containing 10% of plasma from the different adsorption studies in the dynamic model. Untreated plasma from LPS stimulated blood acts as positive control and cell plasma control denotes plasma from blood without LPS stimulation.

	IL-8 [pg/mL]	IL-6 [pg/mL]
Cell plasma control	326 ± 15	231 ± 187
Positive control	6249 ± 858	4056 ± 2124
CG300m	657 ± 72	141 ± 23
HAC	600 ± 53	143 ± 14
CG161c	130 ± 15	66 ± 6
